# Temperature Dependence of Dielectric Properties of Ferroelectric Heterostructures with Domain-Provided Negative Capacitance

**DOI:** 10.3390/nano12010075

**Published:** 2021-12-28

**Authors:** Maksim A. Pavlenko, Yuri A. Tikhonov, Anna G. Razumnaya, Valerii M. Vinokur, Igor A. Lukyanchuk

**Affiliations:** 1 Faculty of Physics, Southern Federal University, 344090 Rostov-on-Don, Russia; makpavlenko@sfedu.ru (M.A.P.); ytihonov@sfedu.ru (Y.A.T.); lukyanc@ferroix.net (I.A.L.); 2 Laboratoire de Physique de la Matière Condensée, Université de Picardie Jules Verne, 80080 Amiens, France; 3 Terra Quantum AG, CH-9400 Rorschach, Switzerland; vmvinokour@gmail.com

**Keywords:** ferroelectrics, heterostructures, domains, negative capacitance

## Abstract

It is well known that the ferroelectric layers in dielectric/ferroelectric/dielectric heterostructures harbor polarization domains resulting in the negative capacitance crucial for manufacturing energy-efficient field-effect transistors. However, the temperature behavior of the characteristic dielectric properties, and, hence, the corresponding behavior of the negative capacitance, are still poorly understood, restraining the technological progress thereof. Here we investigate the temperature-dependent properties of domain structures in the SrTiO_3_/PbTiO_3_/SrTiO_3_ heterostructures and demonstrate that the temperature–thickness phase diagram of the system includes the ferroelectric and paraelectric regions, which exhibit different responses to the applied electric field. Using phase-field modeling and analytical calculations we find the temperature dependence of the dielectric constant of ferroelectric layers and identify the regions of the phase diagram wherein the system demonstrates negative capacitance. We further discuss the optimal routes for implementing negative capacitance in energy-efficient ferroelectric field-effect transistors.

## 1. Introduction

Modern microelectronics is turning to the extensive use of ultrathin ferroelectric films in a broad scope of devices, ranging from ferroelectric random access memory to logic elements and circuits for neuromorphic computers [1,2]. Numerous studies have shown [3] that ferroelectricity, in thin films, persists to nanometer thicknesses, provided that the depolarizing fields arising due to termination of the polarization lines at the film edges are properly reduced. An efficient mechanism for reducing depolarization fields was suggested by [4] and consists of forming a regular array of domains carrying alternating and opposite polarizations. This arrangement leads to the macroscopic neutrality of the depolarizing charges on the surface. However, it has been long believed that such a regular domain structure in ferroelectric films can hardly emerge, due to the screening of the depolarizing field by free charged particles or semiconductor electrons arising from impurities or vacancies. Only recently were domains in thin ferroelectric films discovered and they have become the subject of intense research [5,6,7,8,9]. The domains, predicted in early theoretical studies [5] are studied using the Landau–Kittel approximation, in which the polarization is assumed to be constant in the volume of domains. Subsequently, it has been demonstrated that they have a spatially dependent soft structures [6,10], resembling that of alternating vortices [9].

Superlattices, consisting of ferroelectric and dielectric layers, provide a unique opportunity for studying the behavior of nanodomains in a ferroelectric layer [11] as functions on external conditions, such as the applied field, temperature, and deformation. Due to the dielectric layers protecting the electric neutrality of the ferroelectric layer, the screening of the field by external, trapped charges does not occur. The response of the domain structure to an external applied electric field is of particular interest. As has been shown theoretically and confirmed experimentally, Refs. [12,13,14,15], the average electric field inside the ferroelectric layer, $\langle E_{f}\rangle$, is directed against the average induced polarization of the domain structure, $\langle P_{f}\rangle$. Thus, the effective average dielectric constant of the ferroelectric layer, $\varepsilon_{f}=1+\langle P_{f}\rangle /\varepsilon_{0}\langle E_{f}\rangle$, emergesnegative, and this constitutes the negative capacitance effect viewed as an irreplaceable mean for reducing the power consumption of field-effect transistors [16].

As has been suggested [17,18], the use of negative capacitance in the gate structure of a field-effect transistor may enhance the voltage, controlling the semiconducting channel. Yet, the fact that the negative capacitance owes to the emergence of the domain structure is not adequately appreciated. In addition, the temperature dependence of the susceptibility of the domain structure has not been studied; and, methods for integrating a ferroelectric layer hosting domains into the field effect transistors have not been fully explored.

In this paper, we utilize the phase-field modeling of ferroelectric domains in heterostructures consisting of alternating ultrathin layers of the ferroelectric PbTiO${}_{3}$ (PTO) and dielectric SrTiO${}_{3}$ (STO) to investigate polarization distribution as it depends on temperature and the applied field. Numerical simulations are combined with analytical calculations. The obtained results enable us to explore the behavior of the effective dielectric constant of ferroelectric layers over a wide temperature range. Building on these results, we discuss the possibility of utilizing the domain structures, ensuring negative capacitance for designing energy-efficient ferroelectric transistors and highlighting considerations requiring special care.

## 2. Materials and Methods

Phase-field simulations of PTO/STO heterostructures are carried out using the FEniCS software package, version 2019.2.0.dev0 (https://bitbucket.org/fenics-project, accessed on 8 April 2021, USA) [19]. The appearance of the domain structure is described by the overdamped equation, defining the relaxation of polarization, P, to the state minimizing the system’s free energy, F, to its ground state with time, *t*.

(1) $$-\gamma \frac{\partial P}{\partial t}=\frac{\delta F}{\delta P}.$$

Equation (1) is closed by the Poisson equation, taking into account the electrostatic effects

(2) $$\varepsilon_{0}\varepsilon_{i}\nabla^{2}\varphi =\nabla \cdot P.$$

Here, φ is the electric potential and $\varepsilon_{i}≃10$ [20] is the background dielectric constant due to nonpolar ions. The free energy functional describing the ferroelectric phase transition in a strained PTO has the form of the Ginzburg–Landau functional for a ferroelectric layer subjected to an electric field [21], and is written in the form [22] (the gradient and electrostatic terms are included):

(3) $$\begin{array}{cc} F=\int FdV, & \;F={\left[a_{i}^{*}\left(u_{m},T\right)P_{i}^{2}+a_{ij}^{*}P_{i}^{2}P_{j}^{2}+a_{ijk}P_{i}^{2}P_{j}^{2}P_{k}^{2}\right]}_{i\leq j\leq k}\\ & \;\;\;\;\;\;\;\;\;+\frac{1}{2}G_{ijkl}\left(\partial_{i}P_{j}\right)\left(\partial_{k}P_{l}\right)+\left(\partial_{i}\varphi \right)P_{i}-\frac{1}{2}\varepsilon_{0}\varepsilon_{i}{\left(\nabla \varphi \right)}^{2}, \end{array}$$

where *F* is the free energy density; each of the indices *i*, *j*, *k*, *l* cyclically takes values of 1, 2 or 3 (or *x*, *y*, *z*). The numerical values of the Landau expansion coefficients, $a_{i}^{*}$, $a_{ij}^{*}$, $a_{ijk}$, and gradient energy coefficients, $G_{ijkl}$, for the PTO layers, compressively strained by the STO substrate with lattice mismatch $u_{m}=-0.013$ in the layer plane, are taken from [21,23] and given in Table 1.

The value of the parameter γ, which determines the time scale of relaxation, is not essential for the static case and is assumed be equal to one. In our calculations the coordinate axis, *z*, is directed across the ferroelectric layer, and the *x* and *y* axes lie within the plane of the ferroelectric layer. The coordinate origin is chosen in the middle of the ferroelectric layer. It is assumed that the domains have the structure of stripes, elongated along the *y* axis. For the numerical solution of Equations (1), Newton’s method is used. At each step of calculation, a linear system is solved using the iterative generalized minimum residual method (GMRES) [24,25]. The implementation of the method is described in detail in [26].

## 3. Results

**Figure 1:**
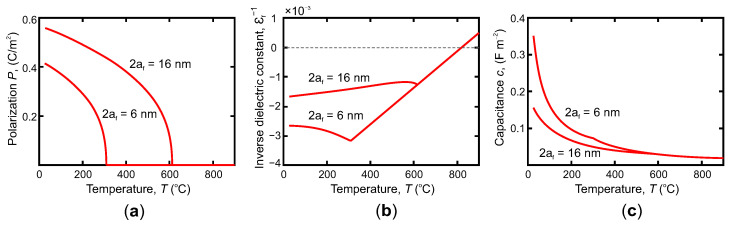
(**a**) Temperature dependence of the average polarization magnitude in the domain structure of ferroelectric PTO layers with thicknesses $a_{f}=6$ nm and $a_{f}=16$ nm; (**b**) temperature dependence of the inverse effective dielectric constant $\varepsilon_{f}^{-1}$ for PTO layers; (**c**) temperature dependence of capacitance (calculated per unit area) for STO/PTO/STO heterostructures with layer thicknesses $a_{f}$/$2a_{f}$/$a_{f}$, where $2a_{f}=6$ nm and $2a_{f}=16$ nm.

Figure 1 shows the calculations for different layer thicknesses’ temperature dependencies on the average polarization, $P={\langle P^{2}\rangle }^{1/2}$, the inverse of average dielectric constant of the ferroelectric layer, $\varepsilon_{f}^{-1}$, and the total capacitance of the heterostructures, *c*, taken per unit area, respectively.

The temperature dependence of the average polarization in the domain state, P(T), shown in panel (a), is typical of second-order phase transitions, where the transition temperature $T_{cd}$ for the 16-nm-thick film being higher than that of the film with 6-nm thickness. This transition is clearly seen as a kink in the dependencies of the inverse dielectric constant upon the temperature, $\varepsilon_{f}^{-1}\left(T\right)$, presented in panel (b). At the same time, the manifestation of the ferroelectric phase transition in the temperature dependence of the total capacitance of the heterostructure, c(T), presented in panel (c), is minor. In the Discussion, we will deliberate these features in detail.

**Figure 2:**
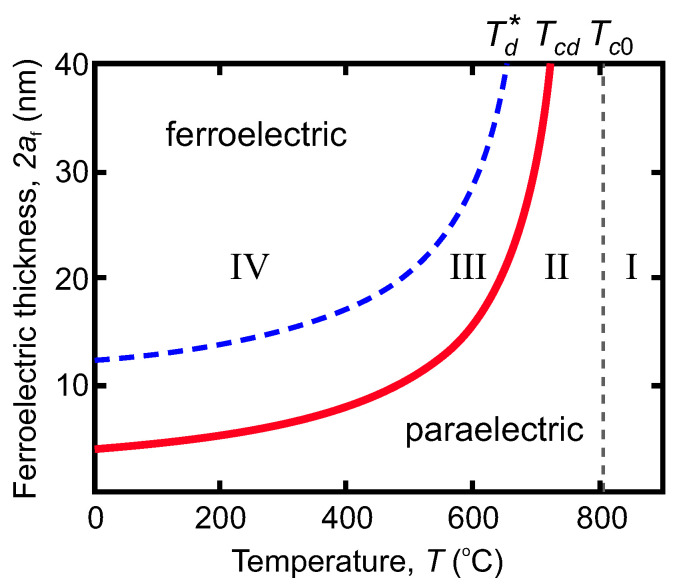
The temperature, *T*—layer thickness, $2a_{f}$, phase diagram of the response of polarization states in a ferroelectric layer to an external field. The roman numbers stand for the states having the different dielectric properties; $T_{c0}$ is the temperature of the transition to the single-domain state when depolarization effects are absent; $T_{cd}$ is the temperature of the transition to the multi-domain state; $T_{d}^{*}$ is the temperature at which soft (vortex) domains acquire the Landau-Kittel domain profile.

The temperature-thickness phase diagram of the ferroelectric layer “*T*-$2a_{f}$” [6], reconstructed on base dielectric and polarization calculations is shown in the Figure 2. It demonstrates four characteristic states, I–IV, revealing the different dielectric response to the applied field. There appear three important characteristic temperatures: $T_{c0}$, the temperature that corresponds to the critical temperature of the bulk short-circuited sample in the absence of depolarization effects (Curie temperature); $T_{cd}$, is the temperature of the transition to the multi-domain state; and $T_{d}^{*}$, the temperature at which the soft (vortex) domains acquire the Landau-Kittel domain profile. At $T_{c0}$, the paraelectric transforms into a so-called incipient ferroelectric state [15,27,28] where the dielectric constant becomes negative, but the depolarization field still suppresses establishing of the global ferroelectricity. The temperature $T_{cd}$ marks the transition from the incipient state to the state where soft ferroelectric domains emerge [11]. Finally, at $T_{d}^{*}$, the blurred domain walls of the soft domains become rigid and narrow, and, as mentioned above, Landau-Kittel domain structure sets in [10]. Respectively, states I and II correspond to the paraelectric phases, while states III and IV correspond to the ferroelectric phases in which domains are formed.

**Figure 3:**
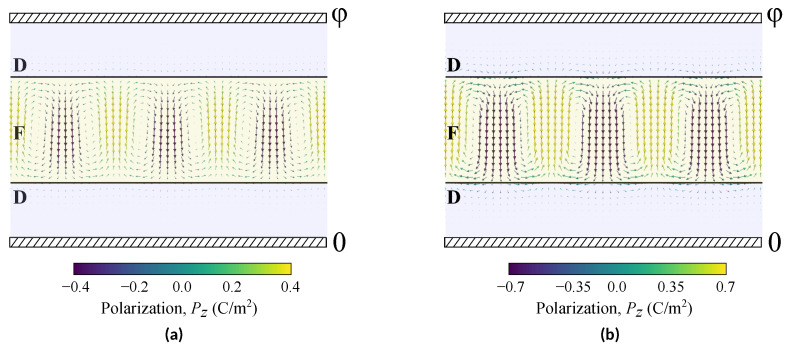
The polarization texture of the dielectric(D)/ferroelectric(F)/dielectric(D) heterostructure STO/PTO/STO with a ferroelectric layer thickness of $2a_{f}=16$ nm (**a**) at temperatures slightly below $T_{cd}$ (soft domains), (**b**) at temperatures below $T_{d}^{*}$ (Landau-Kittel domains).

The Figure 3 depicts polarization textures in a ferroelectric layer with thickness $2a_{f}$ = 16 nm, obtained for different temperatures *T* below $T_{cd}$. The Figure 3a shows the structure of soft domains (state III) arising slightly below $T_{cd}$, and Figure 3b shows the structure of classical Landau-Kittel domains arising below the crossover temperature $T_{d}^{*}$ (state IV).

## 4. Discussion

To describe the dielectric response of the paraelectric and ferroelectric states I–IV, we note that the transition temperature to the multidomain state [6],

(4) $$T_{cd}=\left(1-2\pi {\left(\frac{C}{T_{c0}\varepsilon_{⊥}}\right)}^{1/2}\frac{\xi_{0}}{2a_{f}}\right)T_{c0},$$

is lower than the transition temperature $T_{c0}\approx 800$ ${}^{\circ }$C for a short-circuited (i.e., depolarization field-free) single-domain ferroelectric PTO film, compressively-strained by the STO substrate. Here $C\approx 4.1\times 10^{5}$ ${}^{\circ }$C is the Curie constant of the PTO in the paraelectric phase, $\xi_{0}≃1$ nm is the coherence length, and $\varepsilon_{⊥}≃30$—noncritical dielectric constant transverse to spontaneous polarization in the film plane.

This reduction of $T_{cd}$ is associated with the additional gradient energy required for the domain creation. Therefore, when the temperature drops below $T_{c0}$, the system remains in a supercooled paraelectric state II, and its Curie-type dielectric constant (positive in a conventional paraelectric state I at $T>T_{c0}$)

(5) $$\varepsilon_{p}\left(T\right)=\frac{C}{T-T_{c0}}+\varepsilon_{i},$$

becomes negative below $T_{c0}$. This paraelectric state II with the negative Curie-type dielectric constant exists in the temperature range $T_{cd}<T<T_{c0}$, and then transforms into the so-called soft domain state, denoted as state III in Figure 2. The dielectric constant at the transition point is determined by the relations (4) and (5) as

(6) $$\varepsilon_{p}\left(T_{cd}\right)=-\frac{1}{2\pi }{\left(\varepsilon_{⊥}\frac{C}{T_{c0}}\right)}^{1/2}\frac{2a_{f}}{\xi_{0}}+\varepsilon_{i}.$$

The soft domains structure, shown in Figure 3a, has the profile of a smoothly varying polarization with the period of 2w, which was found from the functional (3) minimization. This profile consists of the spontaneous *z*-oriented polarization, $P_{z}\propto \cos\frac{\pi z}{2a_{f}}\cos\frac{\pi x}{w}$, and the depolarization-field-induced polarization with the predominantly *x*-orientated component $P_{x}=-\varepsilon_{0}\left(\varepsilon_{⊥}-1\right)\partial_{x}\varphi \propto \sin\frac{\pi z}{2a_{f}}\sin\frac{\pi x}{w}$. Here the depolarization potential φ is determined by the Poisson equation $\varepsilon_{0}\varepsilon_{f⊥}\partial_{x}^{2}\varphi +\varepsilon_{0}\varepsilon_{i}\partial_{z}^{2}\varphi =\partial_{z}P_{z}$.

The shown in Figure 3a complete soft polarization profile includes both the spontaneous and induced components, $P=(P_{x},P_{z})$. This texture, also observed experimentally [9], resembles a periodic vortex-antivortex texture and called as vortex phase as well. Negative capacitance in the soft domain phase is due to the locally negative dielectric response of the wide domain wall regions, containing the paraelectric-like phase with P≈0 [16]. Its absolute value decreases slowly with decreasing of the temperature.

Note that the transition to the soft domain phase manifests itself as an insignificant kink in the temperature dependence of the capacitance of the entire heterostructure (Figure 1c), consisting of capacities of the ferroelectric and dielectric layers connected in series, $c^{-1}=c_{f}^{-1}+c_{d}^{-1}$. The constitutive capacitancies, calculated per unit area, are expressed through the dielectric constants of the corresponding layers, $\varepsilon_{f}$ (or $\varepsilon_{p}$ for $T>T_{cd}$) and $\varepsilon_{d}$ as $c_{f}=\varepsilon_{0}\varepsilon_{f}/2a_{f}$ and $c_{d}=\varepsilon_{0}\varepsilon_{d}/2a_{d}$, respectively. Herewith the capacitance of the heterostructure, *c*, is a positive value, which ensures the stability of the system. The plot in Figure 1c presumes that the total layer thicknesses of ferroelectric and dielectric materials are the same, $2a_{f}=2a_{d}$, and that the dielectric constant STO follows the Curie-Weiss law, $\varepsilon_{d}=C_{\mathrm{STO}}/\left(T-T_{\mathrm{STO}}\right)$ where $C_{\mathrm{STO}}\approx 7.83\times 10^{4}$ ${}^{\circ }$C, $T_{\mathrm{STO}}\approx -245$ ${}^{\circ }$C.

Upon the significant decrease in temperature, the domain polarization profiles acquire the form of the classical Landau-Kittel domains, in which the regions of the oppositely-oriented almost uniform polarizations are separated by thin domain walls. The negative sign of the effective dielectric constant arises mostly due to the motion of the domain walls under the influence of the field. Although such a regime is not fully achievable in the nanoscale films (see the phase diagram in Figure 2), the corresponding dielectric constant value calculated in [13],

(7) $$\varepsilon_{f}=\varepsilon_{\|}\left[1-\frac{\pi \varsigma }{4\ln2}{\left(\frac{\varepsilon_{⊥}}{\varepsilon_{\|}}\right)}^{1/2}\frac{2a_{f}}{w}\right],$$

can be considered as the higher limit of the negative dielectric constant, attained at low temperatures. Here $\varsigma =1+\varepsilon_{d}/{\left(\varepsilon_{\|}\varepsilon_{⊥}\right)}^{1/2}$ and $\varepsilon_{\|}\approx C/2T_{c0}$ is the dielectric constants of uniform ferroelectric at low temperatures in the longitudinal to polarization direction.

The slight decrease in the numerically calculated inverse dielectric constant $\varepsilon_{f}^{-1}$ from $-10^{-3}$ to $-1.7\times 10^{-3}$ (i.e., increasing of $\varepsilon_{f}$) with decreasing the temperature below $T_{cd}≃600{\;}^{\circ }$C, shown in Figure 1b for ferroelectric layer with $2a_{f}$ = 16 nm, is in line with the analytical estimations (6) and (7) in which $\varepsilon_{p}$ at $T_{cd}$ is lower then $\varepsilon_{f}$ at $T<T_{cd}$. Surprisingly, however, for thinner layer with $2a_{f}$ = 8 nm the calculated temperature behaviour is the opposite, $\varepsilon_{f}^{-1}$ increases from $-3.2\times 10^{-3}$ to $-2.7\times 10^{-3}$ with decreasing temperature. This difference is attributed to the fact that for such thin films the Landau-Kittel regime is not realized, see phase diagram in Figure 2, and the corresponding approximation (7) does not apply. Note that for the very thin ferroelectric layers the experimentally measured dielectric constant, negative just below $T_{cd}$, becomes again positive at lower temperatures [15]. This reversal behaviour can be explained either by the pinning of domain walls on the structural inhomogeneities, that prevents the domain wall motion, or by specific nonlinear interaction of the STO${}_{3}$ dielectric layer with fringing depolarization field of domains, emerging outside the PTO layer [29].

**Figure 4:**
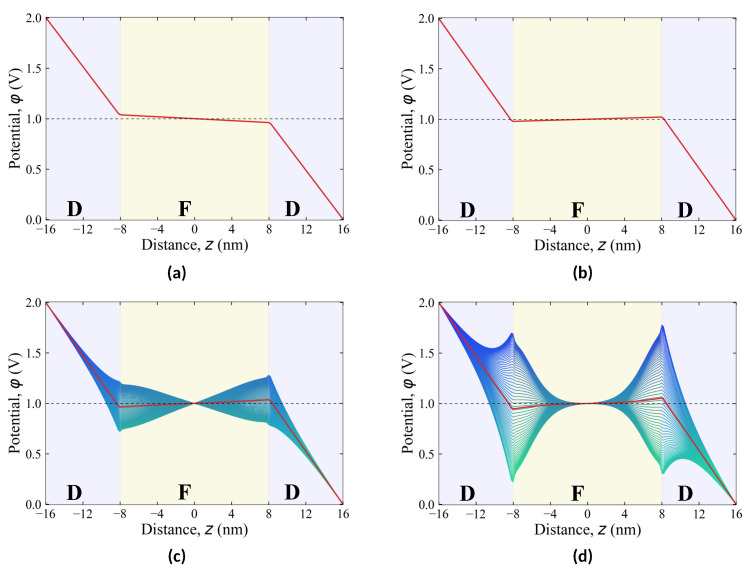
Distribution of the potential φ in the STO/PTO/STO heterostructure depending on its thickness for various states of the ferroelectric PTO layer described in Figure 2: (**a**) state I, (**b**) state II, (**c**) state III, (**d**) state IV.

Figure 4 depicts the *z*-dependence of the electric potential across the heterostructure at the applied voltage *V* = 2 V for states I–IV. In paraelectric states I and II, the induced electric fields inside paraelectric and dielectric layers are uniform, and the corresponding piecewise linear dependence of the potential along *z* is shown in Figure 4a,b by the red line. In ferroelectric states III and IV with domains, the distribution of the induced depolarization electric field is highly non-uniform due to the inhomogeneity of polarization in the domain structure. The spread of the potential dependencies along different lines parallel to *z* in these states around its averaged effective value (piecewise red lines) is shown in Figure 4c,d in blue gamma. Increasing the effective potential in the ferroelectric layer in states II–IV, against the background of the general drop of the potential between the electrodes, corresponds to the described effect of the negative dielectric constant.

Utilizing this effect is expected to help in devising an energy-saving field-effect transistor in which the voltage controlling the conducting channel, formed near the ferroelectric layer, is amplified due to the increase in the effective potential in the ferroelectric layer [17]. However, the domain-induced inhomogeneous distributions of the potential in the ferroelectric layer, also penetrating the dielectric layer over the penetration depth of order *w*, is highly nonuniform in the region of the conducting channel and varies as $\varphi \propto \cos\frac{\pi x}{w}e^{-\pi \delta /w}$, where δ is the distance from the paraelectric–dielectric interface to the channel. As shown in Figure 4c,d, the characteristic spread of the potential near the ferroelectric–dielectric interface, hence, in the channel region, is surprisingly large and is of the order of 1.2 V. Not only does it exceed the amplification of the operating voltage near the conducting channel of order of 0.3 V, arising due to the effect of the ferroelectric negative capacitance, but it is also comparable to the total voltage applied to the transistor.

Such a strong field inhomogeneity can make the conducting channel uncontrollable. Note further that the use of state II with the negative dielectric constant for creating an energy-saving transistor is a technologically challenging task [28]. Although this state does not have an aforementioned inhomogeneity of the field distribution, it has a small gain parameter that is difficult to integrate into the existing semiconductor technologies. Therefore the new routs and architectures should be devised to implement the negative capacitance effect. In particular, the integration of the intermediate floating-gate electrode, compensating the domain-induced field inhomogeneity and allowing for the control of the domain wall displacement [14] holds high technological promise.

## 5. Conclusions

In this work we obtained the temperature dependence of the characteristic dielectric constant of a ferroelectric PTO layer with domains integrated in the STO/PTO/STO heterostructure. We have shown that the inverse dielectric constant of a ferroelectric layer of thickness $2a_{f}$ = 16 nm is negative and decreases from $-10^{-3}$ to $-1.7\times 10^{-3}$ with decreasing temperature in the temperature range of the phase transition temperature $T_{cd}≃600$ ${}^{\circ }$C to room temperature. For the ferroelectric layer with the thickness of 6 nm, the phase transition temperature decreases to 300 ${}^{\circ }$C, and the inverse dielectric constant increases from $-3.2\times 10^{-3}$ to $-2.7\times 10^{-3}$ with decreasing temperature. We demonstrated that the negative capacitance effect associated with a multidomain texture can be used to create energy-efficient field-effect transistors, provided that the domain-induced inhomogeneities of the field in the channel region will be smoothed by the clever architecture of the transistor.

Note that the effect of the negative capacitance takes place in a variety of the nano-sized systems including nanocylinders, nanodots and nanoparticles containing topological structures of polarization, such as vortices [30], skyrmions [26] and Hopfions [31]. The possibility of controlling such formations by an external electric field enables ferroelectric nanostructures to become a basic constituent element of next-generation transistors.

## Figures and Tables

**Table 1:** Numerical values of coefficients in the free energy functional F.

Coefficient	Value	Units
$a_{1}^{*}$, $a_{2}^{*}$	3.8 × 10${}^{5}$ (*T*–479 ${}^{\circ }$C) − 11 × 10${}^{9}u_{m}$	C${}^{-2}$ m${}^{2}$ N${}^{-1}$
$a_{3}^{*}$	3.8 × 10${}^{5}$ (*T*–479 ${}^{\circ }$C) + 9.5 × 10${}^{9}u_{m}$	C${}^{-2}$ m${}^{2}$ N${}^{-1}$
$a_{11}^{*}$, $a_{22}^{*}$	0.42 × 10${}^{9}$	C${}^{-4}$ m${}^{6}$ N
$a_{33}^{*}$	0.05 × 10${}^{9}$	C${}^{-4}$ m${}^{6}$ N
$a_{13}^{*}$, $a_{23}^{*}$	0.45 × 10${}^{9}$	C${}^{-4}$ m${}^{6}$ N
$a_{12}^{*}$	0.73 × 10${}^{9}$	C${}^{-4}$ m${}^{6}$ N
$a_{111}$, $a_{222}$, $a_{333}$	0.26 × 10${}^{9}$	C${}^{-6}$ m${}^{10}$ N
$a_{112}$, $a_{113}$, $a_{223}$	0.61 × 10${}^{9}$	C${}^{-6}$ m${}^{10}$ N
$a_{123}$	−3.7 × 10${}^{9}$	C${}^{-6}$ m${}^{10}$ N
$G_{1111}$	2.77 × 10${}^{-10}$	C${}^{-2}$ m${}^{4}$ N
$G_{1122}$	0.0	C${}^{-2}$ m${}^{4}$ N
$G_{1212}$	1.38 × 10${}^{-10}$	C${}^{-2}$ m${}^{4}$ N

## Data Availability

Data underlying the results presented in this paper are not publicly available at this time but may be obtained from the authors upon reasonable request.
